# Electrophysiological properties and projections of lateral hypothalamic parvalbumin positive neurons

**DOI:** 10.1371/journal.pone.0198991

**Published:** 2018-06-12

**Authors:** Alexandre Kisner, Julia E. Slocomb, Sarah Sarsfield, Maria Laura Zuccoli, Justin Siemian, Jay F. Gupta, Arvind Kumar, Yeka Aponte

**Affiliations:** 1 Neuronal Circuits and Behavior Unit, National Institute on Drug Abuse, Intramural Research Program, National Institutes of Health, Baltimore, Maryland, United States of America; 2 Department of Internal Medicine, Pharmacology and Toxicology Unit, University of Genoa, Genoa, Italy; 3 Department of Computational Science and Technology, School of Electrical Engineering and Computer Science, KTH Royal Institute of Technology, Stockholm, Sweden; 4 The Solomon H. Snyder Department of Neuroscience, Johns Hopkins University School of Medicine, Baltimore, Maryland, United States of America; McGill University Department of Physiology, CANADA

## Abstract

Cracking the cytoarchitectural organization, activity patterns, and neurotransmitter nature of genetically-distinct cell types in the lateral hypothalamus (LH) is fundamental to develop a mechanistic understanding of how activity dynamics within this brain region are generated and operate together through synaptic connections to regulate circuit function. However, the precise mechanisms through which LH circuits orchestrate such dynamics have remained elusive due to the heterogeneity of the intermingled and functionally distinct cell types in this brain region. Here we reveal that a cell type in the mouse LH identified by the expression of the calcium-binding protein parvalbumin (PVALB; LH^PV^) is fast-spiking, releases the excitatory neurotransmitter glutamate, and sends long range projections throughout the brain. Thus, our findings challenge long-standing concepts that define neurons with a fast-spiking phenotype as exclusively GABAergic. Furthermore, we provide for the first time a detailed characterization of the electrophysiological properties of these neurons. Our work identifies LH^PV^ neurons as a novel functional component within the LH glutamatergic circuitry.

## Introduction

The hypothalamus contains a diverse collection of intermingled cell types defined by the expression of classical neurotransmitters and neuropeptides [1, 2]. While extensive research has been done on hypothalamic neurons that express neuropeptides such as agouti-related peptide (AGRP), pro-opiomelanocortin (POMC) [3–6], hypocretin (orexin; HCRT), melanin-concentrating hormone (MCH) [7, 8], or on larger populations defined by neurotransmitter expression (*e*.*g*. vesicular GABA (γ-aminobutyric acid) transporter (SLC32A1 commonly known as VGAT) and vesicular glutamate transporter 2 (SLC17A6 commonly known as VGLUT2)) [9], much less attention has been given to a small collection of neurons expressing the calcium-binding protein parvalbumin (PVALB; PV neurons) in the lateral hypothalamus (LH^PV^ neurons) [10, 11].

Throughout the central nervous system, PV-expressing neurons are typically GABAergic interneurons with fast-spiking characteristics [12] that are necessary and sufficient for the generation of network oscillations in both the neocortex and hippocampus [13, 14]. However, previous studies using immunohistochemistry and gene expression analysis showed that within the LH, PV-expressing neurons colocalize with glutamate in both rats and mice [11, 15]. Nevertheless, quantitative measurements of the numbers of co-expressing neurons and their ability to release glutamate and form functional excitatory synapses have yet to be determined. Furthermore, the fundamental electrophysiological properties, synaptic connections, and the functional roles of LH^PV^ neurons have remained largely uncharacterized. Here, we used a combination of selective targeting, electrophysiology, single-cell RT-qPCR, and *in situ* hybridization assays to characterize the intrinsic properties of LH^PV^ neurons and map their axonal projections.

## Materials and methods

### Animals

All experimental protocols were conducted in accordance with U.S. National Institutes of Health Guidelines for the Care and Use of Laboratory Animals and with the approval of the National Institute on Drug Abuse Animal Care and Use Committee. Two- to five-month-old male and female C57BL/6J (wild-type, Strain 664, The Jackson Laboratory, ME, USA), *Pvalb*^*IREScre*^ (C57BL/6 background, Strain 8069, The Jackson Laboratory), and *Pvalb*^*IREScre*^ crossed with *Rosa26*^*LSL-tdTomato*^ (C57BL/6 background, Strain 7909, The Jackson Laboratory) mice were used in this study. Prior to stereotaxic viral injection, mice were group housed with littermates in temperature- and humidity-controlled rooms with *ad libitum* access to water and rodent chow (PicoLab Rodent Diet 20, 5053 tablet, LabDiet/Land O’Lakes Inc., MO, USA) on a 12 h light/dark cycle.

### Stereotaxic viral injection

For brain slice electrophysiological recordings, two- to five-month-old *Pvalb*^*IREScre*^ heterozygous mice were used. Mice were anesthetized with isoflurane and placed onto a stereotaxic apparatus (David Kopf Instruments, CA, USA). After exposing the skull by a minor incision, small holes (< 1 mm diameter) were drilled bilaterally for virus injection. An adeno-associated virus (rAAV2/rh10-CAG-FLEX-*rev*-ChR2:tdTomato or rAAV2/1-CAG-FLEX-*rev*-CHR2:tdTomato; titer: 8.43×10^12^ genomic copies/ml and 6.86×10^12^ genomic copies/ml, respectively; University of Pennsylvania Gene Therapy Program Vector Core, PA, USA) [3] was injected bilaterally (50−100 nl; rate: 30 nl/min) into the lateral hypothalamus (LH; bregma, −1.80 mm; midline, ±1.40 mm; dorsal surface, −5.40 mm) by a pulled glass pipette (20–30 μm tip diameter) with a micromanipulator (Narishige International USA Inc., NY, USA) controlling the injection speed. Subsequently, the incision was stitched, and mice were individually housed for two to five weeks for post-surgical recovery and viral transduction.

For mapping LH^PV^ axonal projections, eight-week-old *Pvalb*^*IREScre*^ heterozygous mice were bilaterally injected with 30 nl of an adeno-associated virus (rAAV2/9-hEF1α-DIO-hSyn-mCherry; titer: 1.0×10^13^ genomic copies/ml; Massachusetts Institute of Technology Viral Gene Transfer Core, Boston, MA, USA) [16] into the lateral hypothalamus as described above. After surgery, mice were individually housed for six weeks for post-surgical recovery and viral transduction.

### Slice preparation and electrophysiology

After cervical dislocation, mice were decapitated and their brains were rapidly removed and placed into an ice-cold N-methyl-D-glucamine (NMDG)-based slicing solution [17] containing (in mM): 92 NMDG, 20 HEPES, 25 glucose, 30 NaHCO_3_, 1.2 NaH_2_PO_4_, 2.5 KCl, 5 sodium ascorbate, 3 sodium pyruvate, 2 thiourea, 10 MgSO_4_, and 0.5 CaCl_2_, pH 7.4, and osmolality of 304–308 mOsm/kg H_2_O. Acute horizontal brain slices (200–240 μm thick) containing the lateral hypothalamus were obtained using a vibratome (Leica VT1200, Leica Biosystems Inc., IL, USA). Brain slices were transferred to a holding chamber filled with a solution containing (in mM): 92 NaCl, 20 HEPES, 25 glucose, 30 NaHCO_3_, 1.2 NaH_2_PO_4_, 2.5 KCl, 5 sodium ascorbate, 3 sodium pyruvate, 2 thiourea, 1 MgSO_4_, and 2 CaCl_2_ (pH 7.4, 304–308 mOsm/kg H_2_O). For electrophysiological recordings, a single slice was submerged in artificial cerebrospinal fluid (aCSF, in mM: 125 NaCl, 2.5 KCl, 1.25 NaH_2_PO_4_, 1 MgCl_2_ 6H_2_O, 11 glucose, 26 NaHCO_3_, 2.4 CaCl_2_, pH 7.4, and osmolality of 304–308 mOsm/kg H_2_O) in a recording chamber that was continuously perfused by a peristaltic pump (World Precision Instruments, FL, USA), at a flow rate of 1.5 to 2.0 ml/min. All solutions were saturated with 95% O_2_ and 5% CO_2_.

Characterization of the intrinsic electrophysiological properties of lateral hypothalamic parvalbumin (LH^PV^) neurons (*n* = 34) was performed using *Pvalb*^*IREScre*^;*Rosa26*^*LSL-tdTomato*^ mice (*Pvalb*^*cre/+*^;*Rosa26*^*tom/tom*^). Parvalbumin-tdTomato-positive lateral hypothalamic neurons were located in brain slices, first with epifluorescence, followed by infrared differential interference contrast (IR-DIC) optics, using an upright Olympus BX51WI microscope (Olympus Corporation, MA, USA). Whole-cell current-clamp recordings were performed using a MultiClamp 700B amplifier (5 kHz low-pass Bessel filter and 10 kHz digitization using a 1440A Digidata Digitizer) with pClamp 10.3 software (Molecular Devices LLC, CA, USA). Borosilicate glass patch pipettes (2.2–4.5 MΩ) containing (in mM): 135 potassium gluconate, 10 HEPES, 4 KCl, 4 MgATP, 0.3 Na_3_GTP, and 0.2% biocytin (pH adjusted to 7.3 using KOH, and osmolality of 290 mOsm/kg H_2_O). The holding potential was −70 mV and the whole-cell access resistances were ≤ 15 MΩ. All recordings were made at 32 °C.

For channelrhodopsin (ChR2)-assisted circuit mapping (CRACM) of neurons synaptically connected to LH^PV^ neurons, *Pvalb*^*IREScre*^ heterozygous mice were bilaterally injected with an adeno-associated virus into the LH as previously described. Horizontal slices containing the LH from AAV-injected *Pvalb*^*IREScre*^ mice were used and ChR2:tdTomato-containing axons visualized in the LH. Whole-cell voltage-clamp recordings of parvalbumin-negative LH neurons (*n* = 75) were performed using patch pipettes (3.0–4.5 MΩ) containing (in mM): 117 cesium methanesulfonate, 20 HEPES, 0.4 EGTA, 2.8 NaCl, 5 TEA-Cl, 4 Mg-ATP, 0.4 Na-GTP, 3 QX-314, and 0.2% biocytin (pH adjusted to 7.3 using CsOH, and osmolality of 287 mOsm/kg H_2_O). Pipette capacitance was compensated for immediately after the pipette was placed into the recording solution, and whole cell capacitance was compensated for after obtaining whole cell access. To compensate for cell membrane depolarization associated with cesium methanesulfonate-based solutions, holding currents ranging from −15 pA to −40 pA were applied, and the series resistance compensation was at least 70%. Series resistance (10–25 MΩ) was monitored with a –5 mV hyperpolarizing pulse given every 10 s, and only recordings that remained stable over the period of data collection were used. Recorded cells were held at –70 mV and photocurrents were evoked by 1 ms blue (473 nm) light pulses (diode-pumped solid-state laser; OptoEngine LLC, UT, USA) delivered at a frequency of 0.1 Hz to determine synaptic connectivity. Light-evoked glutamatergic currents were blocked by perfusing the ionotropic glutamate receptor antagonist, 6,7-dinitroquinoxaline-2,3-dione (DNQX; 10 μM) and D-(-)-2-amino-5-phosphonopentanoic acid (D-AP5; 50 μM). The liquid junction potential for these measurements was not corrected. All recordings were made at 32 °C. All chemicals were obtained from Sigma-Aldrich (MO, USA) or Tocris Bioscience (Bristol, UK).

### Electrophysiological analysis

Intrinsic membrane properties of LH^PV^ neurons were characterized in current-clamp configuration as previously described [18, 19]. Briefly, the resting membrane potential (V_rmp_) and capacitance of the cell membrane (C_mem_) were measured directly after obtaining whole-cell configuration. The action potential (AP) properties were determined from the first AP evoked by applying 500 ms depolarizing current steps in a range of 20–100 pA in 20 pA increments. The respective AP parameters were determined from the first evoked AP. Both the action potential (AP) and fast after-hyperpolarization (fAHP) amplitudes were determined relative to the AP threshold (*i*.*e*. membrane potential (V_m_) at which dV_m_/dt first reached 20 V/s). The fAHP latency was determined as the time difference between the AP threshold level and the largest fAHP peak. AP latency was measured as the time difference between the current onset time and the time when the AP peak was reached, while the AP half-width (HW) was determined as the halfway duration between AP peak and the AP threshold level. AP broadening was measured at twice current amplitude of the first evoked AP and calculated according to (HW2 –HW1)/HW1, where HW1 and HW2 correspond to the HW of the first and second APs, respectively. Further membrane properties and firing rate were determined by applying 500 ms current step injections ranging from −100 pA to 1500 pA in 20–50 pA increments. Input resistance (R_in_) was calculated from the slope of a linear regression fit to the steady-state voltage-current relation using a range of hyperpolarizing current steps. The membrane time constant (τ) was determined by single exponential fit to 63.2% of the rising phase of a mean voltage response to a −100 pA current step. The sag ratio was determined according to V_steady_/V_hyp_, where V_steady_ and V_hyp_ correspond to the voltage response measured at the end of a −100 pA hyperpolarizing current step and the hyperpolarization peak, respectively. The maximal firing frequency was determined as the spike frequency response to the largest depolarizing current step (500 ms) below that where AP failures were observed. To generate the *I–f* curves, we measured the firing rate by counting the number of spikes elicited in response to step depolarizing current injections during a 500 ms window. Note that each neuron was tested for a subset of current amplitudes from 0−1200 pA.

### Immunohistochemistry

Mice were deeply anesthetized with isoflurane and transcardially perfused with phosphate buffered saline (1× PBS) followed by 4% paraformaldehyde (PFA) in 1× PBS. Whole brains were removed and post-fixed in 4% PFA for 2−4 h at 4 °C and subsequently transferred to 1× PBS for storage at 4 °C. Horizontal brain sections (50–70 μm thick) containing the LH were collected in 1× PBS using a vibratome (Leica VT1200), and freely floating slices were immunostained for parvalbumin. First, slices were incubated in a solution of 1× PBS/0.3% Triton X-100 with 10% normal donkey serum (NDS) for 1 h at room temperature. Slices were then incubated with either rabbit anti-parvalbumin antibody (1:500 PV25 or PV27; Swant, Marly, Switzerland) or rabbit anti-DsRed antibody (1:1000; Takara Bio USA, Inc., CA, USA) in a solution of 1× PBS/0.3% Triton X-100/2% NDS for 14–18 h at 4 °C. Slices were washed in 1× PBS (4 × 10 min each) and then incubated for 3 h with secondary antibody (1:500 donkey anti-rabbit-AlexaFluor 488 or AlexaFluor 647, Invitrogen, CA, USA) in 1× PBS/0.3% Triton X-100/2% NDS at room temperature. Following PBS washes (4 × 10 min each), slices were mounted onto Superfrost Plus glass slides (VWR International, PA, USA) and coverslipped with DAPI-Fluoromount-G aqueous mounting medium (Electron Microscopy Sciences, PA, USA).

Images were taken with an AxioZoom.V16 fluorescence microscope and z-stacks were collected using an LSM700 laser scanning confocal microscope (Carl Zeiss Microscopy LLC, NY, USA). Stacks were imported into Vaa3D 3D visualization-assisted analysis software [20], and fluorescent neurons were counted bilaterally from every section (*n* = 3 mice).

### Fluorescence *in situ* hybridization

Following cervical dislocation, wildtype mice were decapitated, and brains were dissected and rapidly frozen in −80 °C isopentane, then subsequently stored at −80 °C. Horizontal cryosections (16 μm) containing the lateral hypothalamus were sliced using a Leica CM3050 S cryostat (Leica Biosystems Inc.) and sections collected onto Superfrost Plus glass slides (VWR International). Slides were stored at −80 °C prior to processing. Fluorescent *in situ* hybridization was performed using the RNAscope^®^ Multiplex Fluorescent Assay for fresh frozen tissue (Advanced Cell Diagnostics Inc., CA, USA). Briefly, sections were fixed in 4% PFA in PBS, dehydrated by ethanol series, and treated with Protease IV. Sections were incubated with target probes for mouse parvalbumin (*Pvalb*, accession number NM_013645.3, target region aa2-885), vesicular glutamate transporter 2 (*Slc17a6 (Vglut2)*, accession number NM_080853.3, target region aa1986-2998), and vesicular GABA transporter (*Slc32a1 (Vgat)*, accession number NM_009508.2, target region aa894-2037). After hybridization, a series of signal amplification steps (Amp1, Amp2, and Amp3) were performed per kit protocol followed by incubation with labels (Amp4B) for fluorescent visualization of each probe: *Pvalb* (Alexa488), *Vglut2* (Atto550), and *Vgat* (Atto647). Slides were counterstained with DAPI and coverslipped with Fluoromount-G aqueous mounting medium (Electron Microscopy Systems). Z-stack images were obtained using an LSM700 confocal microscope (Carl Zeiss Microscopy). LH^PV^ neurons were manually assessed for co-expression of *Pvalb* with *Vglut2* or *Vgat*.

### Radioactive *in situ* hybridization and immunohistochemistry

Wildtype mice were deeply anesthetized with isoflurane and transcardially perfused with 0.1 M phosphate buffer (PB) followed by 4% PFA in 0.1 M PB. Whole brains were removed and post-fixed in 4% PFA/PB for 2 h at 4 °C. Samples were washed with 0.1 M PB (2 × 30 min each) at 4 °C, and then transferred to 18% sucrose in 0.1 M PB. Free-floating coronal cryosections (14 μm) were sliced using a Leica CM3050 S cryostat. *In situ* hybridization was performed as previously detailed [21, 22]. Steps are at room temperature unless otherwise noted. Sections were incubated for 3 × 10 min in 0.1 M PB/0.5% Triton X-100, rinsed with 0.1 M PB (3 × 10 min), treated with 0.2 N HCl for 15 min, rinsed with 0.1 M PB (3 × 10 min), and then acetylated in 0.25% acetic anhydride/0.1 M triethanolamine pH 8.0 for 10 min. Sections were rinsed for 3 × 10 min with 0.1 M PB, fixed with 4% PFA/PB for 10 min, washed again with 0.1 M PB (3 × 10 min), and then prehybridized for 2 h at 55 °C. Sections were hybridized for 16 h at 55 °C with [^35^S]- and [^33^P]-labeled (10^7^ c.p.m./ml) antisense probe for either *Vglut2* (nucleotides 317–2357; Accession Number NM_053427) or *Vgat* (nucleotides 1–2814, Accession Number BC052020). After hybridization, sections were incubated in 2× SSC buffer/10 mM β-mercaptoethanol (BME) for 30 min at room temperature. Sections were next treated with 5 μg/ml RNase A in 10 mM Tris-HCl pH 7.9/10 mM NaCl/0.1 mM EDTA for 1 h at 37 °C, washed in 0.5× SSC/50% formamide/10 mM BME/0.5% sarkosyl for 2 h at 55 °C, washed in 0.1× SSC/10 mM BME/0.5% sarkosyl for 1 h at 60 °C, and rinsed with 0.1 M PB (3× 10 min) prior to parvalbumin immunolabeling.

For immunohistochemistry, sections were blocked with 0.1 M PB/4% bovine serum albumin (BSA)/3% Triton X-100 for 1 h. Sections were incubated with goat anti-parvalbumin antibody (1:1000 PVG-213, Swant) in block solution overnight at 4 °C. After washing with 0.1 M PB (3 × 10 min), sections were incubated with biotinylated anti-goat IgG secondary antibody (1:200) in block solution, washed with 0.1 M PB (3 × 10 min), incubated for 1 h in avidin-biotinylated horseradish peroxidase (1:200, ABC kit; Vector Laboratories, CA, USA), rinsed with 0.1 M PB (3 × 10 min), and developed with 0.05% 3,3-diaminobenzidine-4 HCl (DAB)/0.003% hydrogen peroxide/0.1 M PB for 10 min. Sections were washed with 0.1 M PB (3 × 10 min), mounted onto coated slides, dipped in Ilford K5 nuclear tract emulsion (Harman Technology Ltd, TX, USA), and exposed in the dark at 4 °C for four weeks prior to development.

Sections were imaged with brightfield and epiluminescence microscopy using a Leica DMR microscope with a 20× objective and cellSens Standard v1.11 software (Olympus Corporation). Neurons observed within the LH^PV^ region were manually assessed for the co-expression of *Vglut2* mRNA or *Vgat* mRNA with anti-parvalbumin immunolabeled cells. Brightfield was used to determine whether a parvalbumin-immunolabeled (brown DAB product) neuron contained the aggregates of silver grains for *Vglut2* mRNA or *Vgat* mRNA, which were viewed under epiluminescence.

### Single-cell gene expression profiling and analysis

Single-cell cytoplasm harvesting was performed as previously described [23]. Brain slices were prepared from *Pvalb*^*cre/+*^;*Rosa26*^*tom/tom*^ mice as described above. First, the fast-spiking firing pattern of an LH^PV^ tdTomato-positive neuron was recorded during whole-cell configuration. Subsequently, the cytoplasm of the recorded neuron was harvested into the recording pipette. The stability of the gigaseal (*i*.*e*. seal between the neuron and the pipette) was constantly monitored to avoid extracellular contamination. The total recording time and harvesting of the intracellular content did not exceed more than 4 min. The content of the pipette tip containing the harvested cytoplasm was then expelled into an RNase-free PCR tube. For positive control samples, we patched fast-spiking parvalbumin-expressing (tdTomato-positive) basket cells in the hippocampus and performed the same procedure as described above. Single-cell extracts were processed using the Ambion^®^ Single Cell-to-CT Kit (Thermo Fisher Scientific, MA, USA). Briefly, samples were incubated in lysis solution with DNase I. Reverse transcription was then performed followed by preamplification of all target genes using pooled TaqMan Gene Expression Assays (Thermo Fisher Scientific). Target genes in LH^PV^ cells were detected using the recommended best coverage assays for *Pvalb* (Mm00443100_m1), *Slc17a6* (*Vglut2*, Mm00499876_m1), *Slc32a1* (*Vgat*, Mm00494138_m1), *Kcnc1* (*Kv3*.*1*, Mm00657708_m1), *Kcnc2* (*Kv3*.*2*, Mm01234232_m1), and *Hcn2* (Mm00468538_m1). qPCR was performed in 10 μl reactions in 96-well plates using an Applied Biosystems 7500 Fast Real-Time PCR System (Invitrogen, CA, USA) with the following cycling parameters: (1) 50 °C for 2 min, (2) 95 °C for 10 min, and (3) 40 repeats of 95 °C for 15 s, 60 °C for 1 min. Reactions included TaqMan Gene Expression Master Mix, TaqMan probe, and cDNA according to manufacturer’s protocol (Thermo Fisher Scientific). Each plate was run with a negative control (no cDNA template) and a positive control (hippocampal basket cell positive for *Pvalb*, *Vgat*, *Kv3*.*1*, *Kv3*.*2*, and *Hcn2*). Technical replicates (triplicate) were performed for each sample-gene combination. Cycle threshold (Ct) values were determined using Applied Biosystems 7500 v2.0.6 software (Invitrogen). Amplification Ct values higher than 37 and samples lacking any amplification curves were designated below the limit of detection. Three out of eight LH^PV^ cells lacked amplification curves for *Kv3*.*2*. These samples were excluded from the *Kv3*.*2* analysis. For all other target genes, *n* = 8 cells. Gene expression was normalized to *Pvalb* and *Vglut2* using the 2^−ΔΔCt^ method [24].

### Statistical analysis

Data are reported as mean ± s.e.m. or mean ± s.d. unless otherwise noted. Individual data points are shown for qPCR. Data from electrophysiological recordings were analyzed with Clampfit v10.6 (Molecular Devices LLC, CA, USA), Origin Pro v9.2 (OriginLab Corporation, MA, USA) and MATLAB R2015A (The MathWorks Inc., MA, USA). Analyses were performed using the Analysis ToolPak of Microsoft *Excel* 2016 and OriginPro v9.2.

## Results

### LH^PV^ neurons display a fast-spiking action potential phenotype

**Fig 1:**
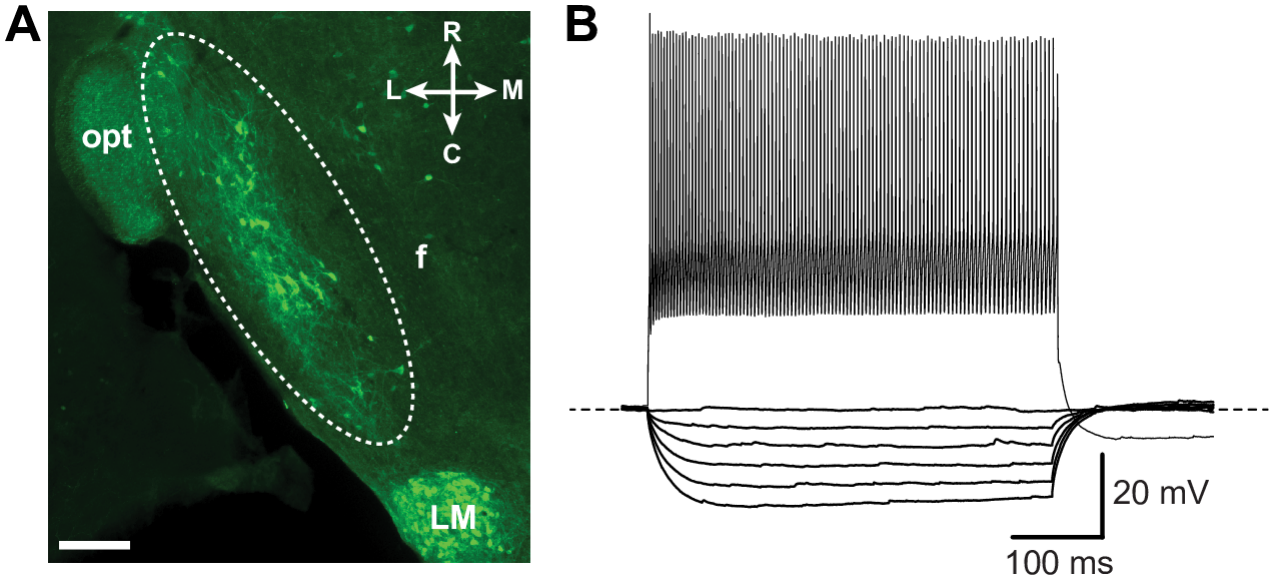
LH^PV^ neurons exhibit fast-spiking characteristics. (A) Immunohistochemical identification of parvalbumin-expressing neurons in a horizontal section of the mouse LH. Dotted lines highlight a cluster of immuno-positive LH^PV^ neurons (green). Scale bar, 200 μm. (B) Representative traces and firing pattern of a fast-spiking LH^PV^ neuron in response to step hyperpolarizing (bottom traces; from −100 to 0 pA) and depolarizing current injections (upper traces; 900 pA) during a 500 ms pulse. Resting membrane potential (V_rmp_ = −66 mV) and maximal firing frequency 264 Hz. Abbreviations, fornix (f), optic tract (opt), lateral mammillary nucleus (LM), lateral (L), medial (M), rostral (R), and caudal (C).

**Fig 2:**
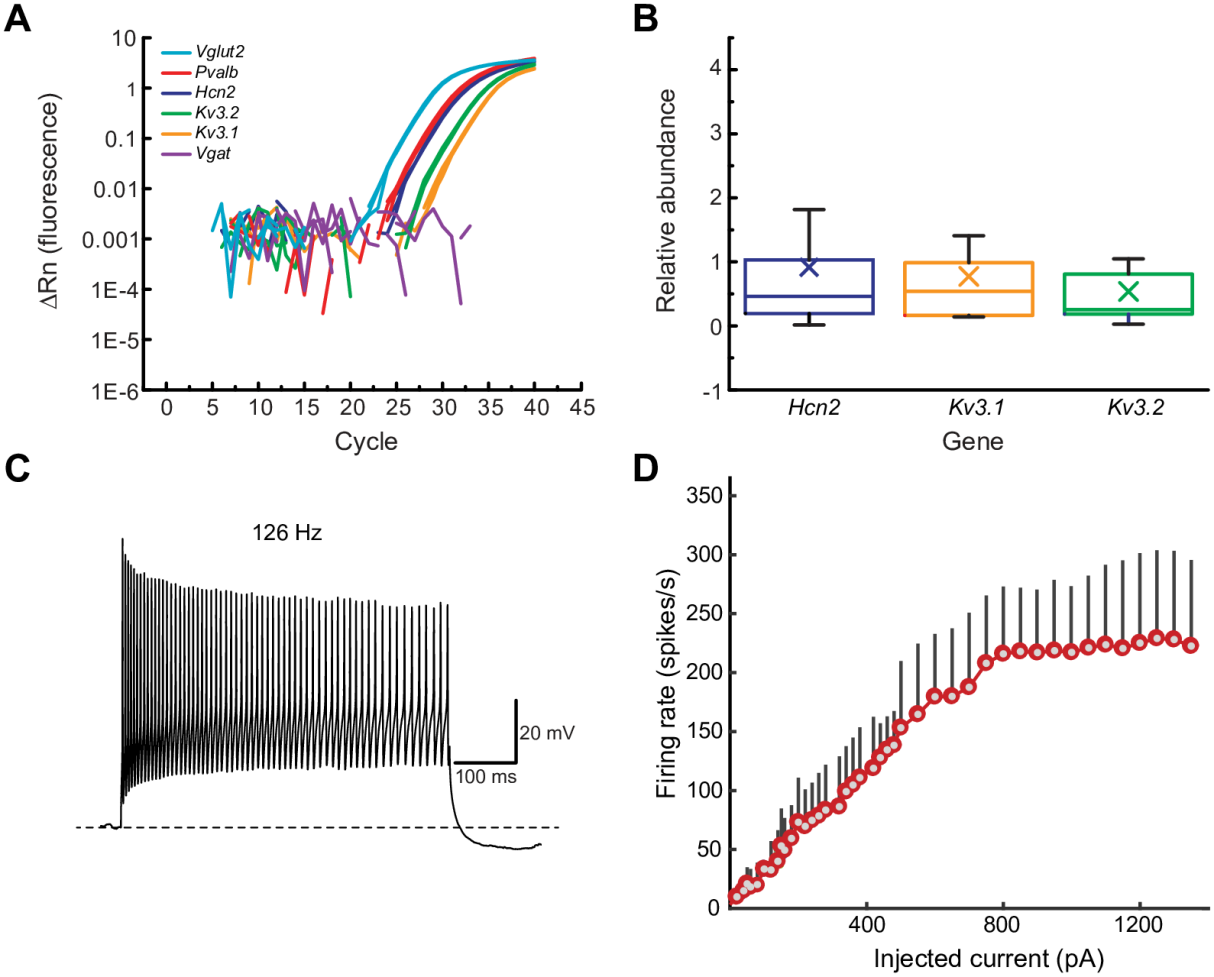
Molecular and electrophysiological characterization of LH^PV^ neurons. (A) Detection of *Kv3*.*1*, *Kv3*.*2*, and *Hcn2* subunit genes by RT-qPCR analysis after harvesting the cytoplasm from single LH^PV^ neurons. Representative amplification plot displayed. Note that cells were *Pvalb*^+^/*Vglut2*^+^/*Vgat*^−^. (B) Relative abundance of *Kv3*.*1*, *Kv3*.*2*, and *Hcn2* in single LH^PV^ neurons. Box plots show mean (×), median, quartiles (boxes), and s.e.m. (whiskers). Cycle threshold (Ct), relative abundance values, and sample sizes are explained in Methods. (C) Representative firing pattern of a fast-spiking LH^PV^ neuron that displays spike frequency accommodation and amplitude attenuation during large depolarizing current injections (500 pA, 500 ms pulses). Note decreases in firing frequency and amplitude during the last 100 ms of the pulse. Dotted line denotes resting membrane potential (V_rmp_ = –63 mV). (D) Firing rate of LH^PV^ neurons in response to current injection (*I–f* curves) during 500 ms pulses. The red/gray dots show the average firing rate of the LH^PV^ neurons and the standard deviation is indicated by the black vertical bar (*n* = 34).

We first characterized the electrophysiological properties of LH^PV^ neurons under current-clamp conditions. These neurons form a compact and small cluster in the LH medial to the optic tract (340 ± 10 neurons, *n* = 3 mice; bilateral; Fig 1A), and the number of cells was consistent with previous studies estimating ~400 PV-immunoreactive neurons in the LH of mice [11]. We performed whole-cell current clamp recordings from LH^PV^ neurons (*n* = 34) identified by tdTomato fluorescence in brain slices sectioned horizontally from *Pvalb*^*IREScre*^;*Rosa26*^*LSL-tdTomato*^ mice and observed that these neurons fired action potentials at high-frequency and with little accommodation in response to depolarizing current injections (Fig 1B). This fast-spiking action potential phenotype and other electrophysiological characteristics such as the resting membrane potential (V_rmp_), action potential half-width (AP_HW_), and the maximal firing frequency (Table 1) are similar to the properties of both hippocampal and neocortical PV-positive GABAergic interneurons [13, 14]. Previous studies showed that fast-spiking interneurons can be characterized by the expression of a specific combination of ion channels that confer these electrophysiological properties as for example delayed rectifier voltage-gated potassium channels KCNC1 (KV3.1) and KCNC2 (KV3.2) and hyperpolarization-activated cation channels (HCNs) [12]. Therefore, we sought to determine whether the expression of these channels is similar in LH^PV^ neurons. Single-cell RT-qPCR analysis revealed that LH^PV^ neurons express *Kv3*.*1*, *Kv3*.*2*, and *Hcn2* subunit genes with comparable relative abundance (Fig 2A and 2B). Together, these results demonstrate that LH^PV^ neurons are equipped with ion channels that are implicated in setting precise pacing of spiking activity to minimize accommodation during prolonged depolarization with some electrophysiological diversity (Fig 2C and 2D) possibly attributable to the variations in the relative contributions of specific ion channels.

**Table 1:** Electrophysiological properties of LH^PV^ neurons.

*Electrophysiological Property*	
V_rmp_ (mV)	−65.6 ± 1.2
C_mem_ (pF)	40.2 ± 2.5
R_in_ (MΩ)	327.3 ± 29.6
τ_m_ (ms)	21.3 ± 1.8
Sag ratio	0.96 ± 0.004
AP threshold (mV)	−40.5 ± 1.1
AP latency (ms)	49.5 ± 6.7
AP amplitude (mV)	63.4 ± 1.3
AP_HW_ (ms)	0.45 ± 0.02
fAHP amplitude (mV)	21.9 ± 0.8
fAHP latency (ms)	1.97 ± 0.13
AP broadening	0.06 ± 0.01
Maximal firing frequency (Hz)	193.6 ± 10.3

Descriptions of electrophysiological properties (*n* = 34 neurons) are explained in Materials and Methods. Data are reported as mean ± s.e.m.

### LH^PV^ neurons release the excitatory neurotransmitter glutamate and provide excitatory inputs onto neurons within the LH

**Fig 3:**
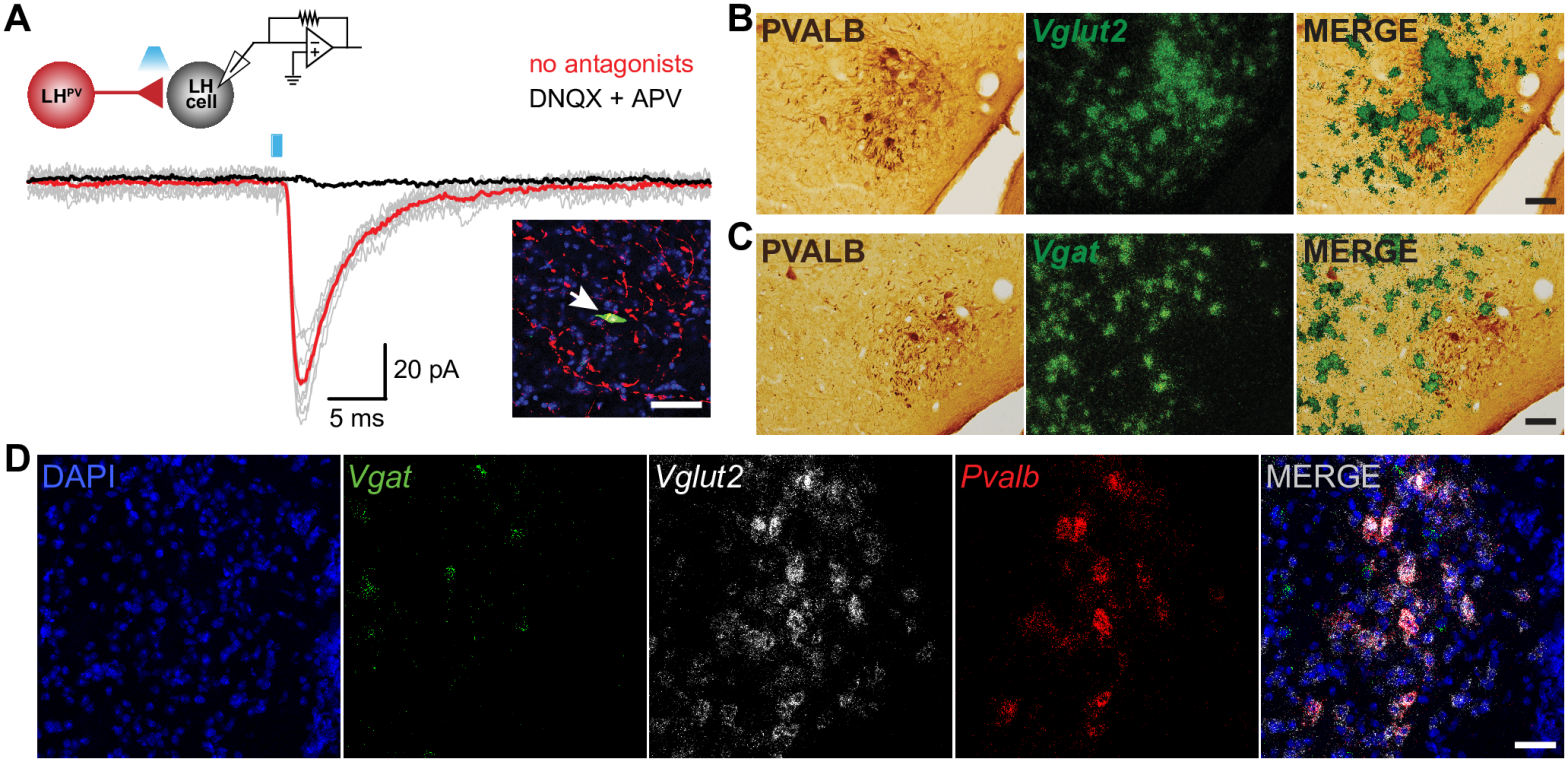
LH^PV^ neurons are *Vglut2*-positive and release glutamate. (A) Traces of excitatory postsynaptic currents (EPSCs; gray) evoked by photostimulation (1 ms light pulses) of LH^PV^-ChR2^+^ neurons before and after bath application of DNQX and APV (black trace; AMPA-R and NMDA-R antagonists). Red and black traces are the average of ten consecutive sweeps. LH neuron was held at −70 mV. Note schematic of ChR2-assisted circuit mapping (inset; upper left) from LH^PV^-ChR2^+^ neuron (red) onto a postsynaptic lateral hypothalamic neuron (gray) as well as arrow indicating the recorded postsynaptic LH neuron (inset; bottom right) filled with biocytin (green) surrounded by ChR2:tdTomato-expressing LH^PV^ dendrites/axons (red). Scale bar, 50 μm. (B-C) Coronal sections showing that parvalbumin immunoreactive cells (brown; left panel) mainly colocalize with *Vglut2* mRNA (95%; green grain aggregates; right panel) but not with *Vgat* mRNA (5%; green grain aggregates; right panel). Scale bars, 50 μm. (D) Fluorescent *in situ* hybridization assay for *Vgat* (green), *Vglut2* (white), and *Pvalb* (red) with DAPI (blue) counterstain. *Pvalb* mRNA was predominantly detected in neurons that express *Vglut2* (95% *Pvalb*^+^*/Vglut2*^+^; 5% *Pvalb*^+^*/Vgat*^+^) Scale bar, 50 μm.

**Fig 4:**
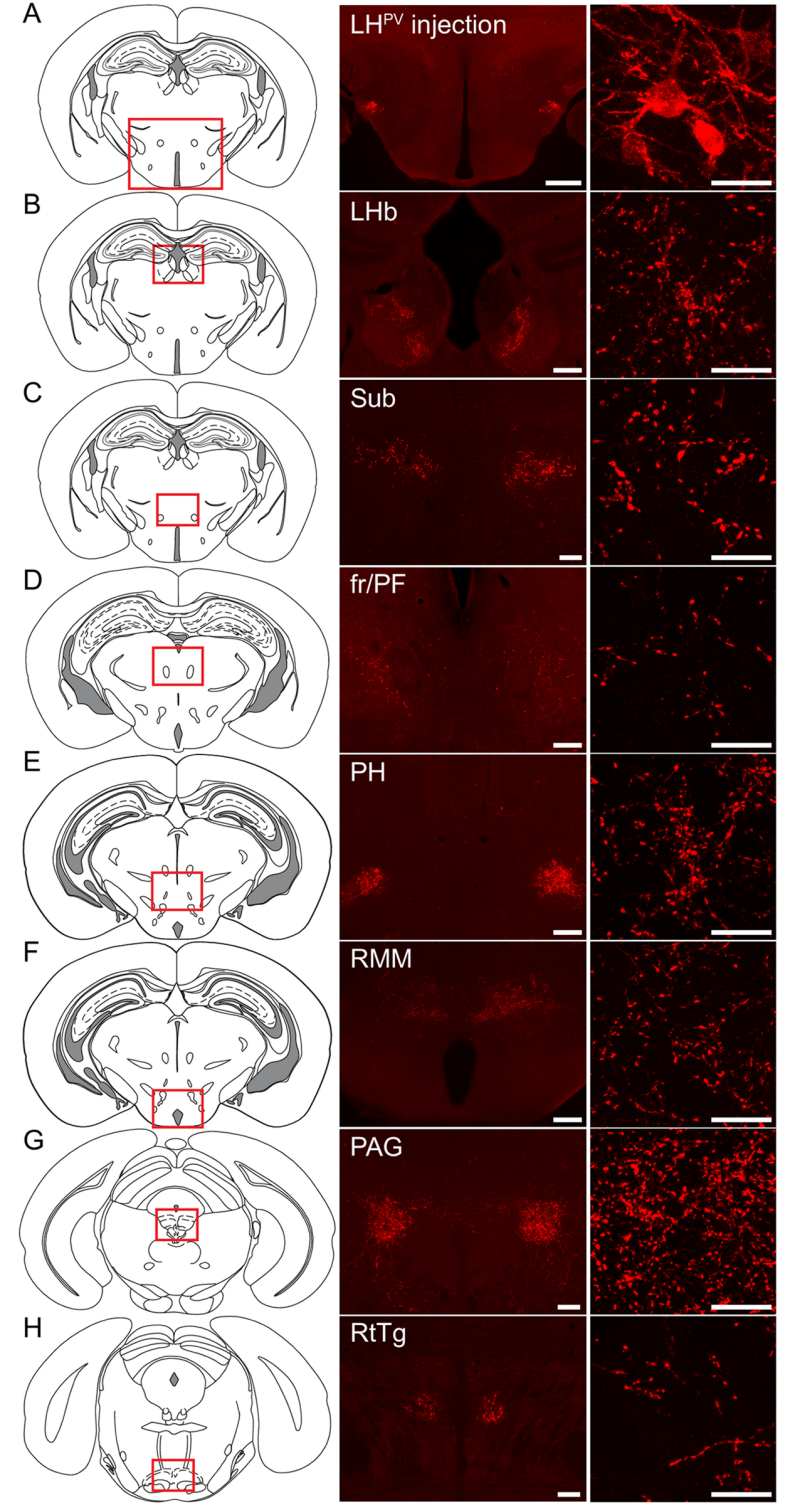
Axonal projections of LH^PV^ neurons. (A) Schematic and representative image depicting a bilateral injection of the Cre recombinase-dependent viral vector for anterograde tracing (rAAV2/9-hEf1α-DIO-synaptophysin-mCherry) into the lateral hypothalamus (LH) of a *Pvalb*^*IREScre*^ mouse. Scale bar 500 μm. Representative images of projections to (B) the lateral habenula (LHb), (C) the submedius thalamic nucleus (Sub), (D) the parafascicular thalamic nucleus (PF) surrounding the fasciculus retroflexus (fr), (E) the posterior hypothalamus (PH), (F) the retromamillary nucleus (RMM), (G) the periaqueductal gray (PAG), and (H) the reticulotegmental nucleus of the pons (RtTg). (B-D-E-F) Scale bars (low magnification), 200 μm; Scale bars (high magnification), 25 μm (C-G-H) Scale bars (low magnification), 100 μm; Scale bars (high magnification), 25 μm. (*n* = 3 mice). Schematic images modified from Franklin KBJ & Paxinos G (2013) [27].

Thus far, our results indicate that LH^PV^ neurons have several features in common with PV-positive GABAergic interneurons in other brain regions. Therefore, we next sought to determine whether LH^PV^ neurons are GABAergic. Surprisingly, previous studies have shown that parvalbumin can be found colocalized with glutamate immunohistochemically in both, rat and mouse LH [11, 15]. However, a quantification of the number of LH^PV^ neurons expressing specific markers for glutamate and their ability to release the neurotransmitter and form functional synapses have not been determined. Therefore, we used several approaches to determine the neurotransmitter used by LH^PV^ neurons. We first performed channelrhodopsin (ChR2)-assisted circuit mapping (CRACM) [25, 26] to examine whether LH^PV^ neurons are synaptically connected to other cells within the LH (Fig 3A). We stereotaxically injected a Cre recombinase-dependent viral vector [26] bilaterally into the LH of *Pvalb*^*IREScre*^ transgenic mice to target channelrhodopsin-2 (ChR2) fused to the fluorophore tdTomato (ChR2:tdTomato) specifically to LH^PV^ neurons. We next performed whole-cell recordings from individual PV-negative neurons within the LH (*n* = 75) under voltage-clamp configuration (Fig 3A) and observed that photostimulation of ChR2-expressing LH^PV^ neurons and axons evoked excitatory postsynaptic currents (EPSCs; 69.0 ± 5.0 pA) in synaptically connected LH neurons (*n* = 13; 17.33% connected). These EPSCs were also blocked by bath applied selective antagonists of glutamate receptors (2.5 ± 0.4 pA). This demonstrates that rather than releasing GABA, LH^PV^ neurons release the excitatory neurotransmitter glutamate. To determine whether LH^PV^ neurons contained markers of glutamate or GABA neurons, we performed *in situ* hybridization assays to measure the expression of *Slc17a6* (*Vglut2*; vesicular glutamate transporter 2) and *Slc32a1* (*Vgat*; vesicular GABA transporter) in these neurons (Fig 3B and 3D). We observed that *Pvalb* mRNA was predominantly detected in neurons that express *Vglut2* (95% *Pvalb*^+^*/Vglut2*^+^; 5% *Pvalb*^+^*/Vgat*^+^; *n* = 4). Together, these results describe a cluster of glutamatergic fast-spiking LH^PV^ neurons that provide excitatory inputs to neurons within the LH and send long range projections throughout the brain (*n* = 3, Fig 4A–4H).

## Discussion

The roles of specific, genetically-distinct lateral hypothalamic neuronal types in regulating circuit function have not been thoroughly identified. Here, we performed for the first time a comprehensive characterization of the electrophysiological properties of a subclass of LH neurons known as LH^PV^ cells, and we implicate them in the regulation of activity dynamics within the LH.

We find that LH^PV^ neurons exhibit a fast-spiking action potential phenotype and have electrophysiological characteristics similar to hippocampal and neocortical parvalbumin-positive GABAergic interneurons [13, 14]. Moreover, like those neurons, we reveal that LH^PV^ cells express a combination of ion channels that confer these electrophysiological properties [12]. Our single-cell RT-qPCR analysis demonstrates that LH^PV^ neurons are equipped with ion channels that are implicated in setting and regulating fast-spiking activity [12]. Interestingly, heterogeneous firing patterns were observed in a preliminary study attempting to record spontaneous activity from LH^PV^ neurons extracellularly in anaesthetized rats [28]. However, these experiments were performed with traditional extracellular electrophysiology methods in which an intrinsic limitation is the accurate identification of the recorded cell types. Therefore, further work using a combination of optogenetics and electrophysiological methods is needed to determine the activity patterns of LH^PV^ neurons *in vivo* and during behavior.

In the LH, immunohistochemical colocalization of parvalbumin and glutamate has been reported [11, 15]. However, here we provide a quantitative measurement of the number of glutamatergic LH^PV^ neurons. Furthermore, we demonstrate that these cells release glutamate and provide excitatory control of local neuronal circuits within the LH. Remarkably, our findings and those of others [29, 30], challenge long-standing conceptualizations that fast-spiking neurons are exclusively GABAergic, suggesting conservation of the fast-spiking phenotype across at least two neurotransmitter systems.

Our results showing that LH^PV^ neurons send long range axonal projections to several brain regions, including the lateral habenula (LHb), submedius thalamic nucleus (Sub), retromamillary nucleus (RMM), and periaqueductal gray (PAG) provide further support for the idea that the LH and its diverse collection of genetically-distinct cell types are crucial for orchestrating a variety of motivated behaviors. In particular, the Sub has been implicated in learning and decision making [31], and the PAG mediates fear-related freezing behavior [32]. Interestingly, a recent study showed that glutamatergic lateral hypothalamic inputs to the LHb regulate reward and feeding-related behaviors [33]. However, as all glutamatergic neurons in the LH were targeted during the study, the contribution of specific genetically-distinct glutamatergic LH neurons is yet to be determined. Thus, it is possible that LH^PV^ neurons regulate circuit function through connections in the LHb. Further experiments using a combination of optogenetics and behavioral assays are needed to determine whether LH^PV^ neurons regulate reward-related behaviors. Similarly, further work is needed to determine functional inputs to LH^PV^ neurons. Inhibitory inputs specifically innervating and suppressing glutamatergic neurons in the LH have been shown to promote feeding behaviors [9]. Based on what we have just described, our work identifies LH^PV^ neurons as a novel functional component within the LH glutamatergic circuitry. Thus, these cells could possibly regulate consummatory and appetitive behaviors.

In summary, our work has revealed that LH^PV^ neurons form functional excitatory synapses with other lateral hypothalamic neurons, which directly implicates LH^PV^ neurons in the regulation of activity dynamics within the LH. Thus, our findings will serve as a basis for future models of lateral hypothalamic circuitry that regulate behaviors essential for survival.

## References

[1] van den Pol AN. Weighing the role of hypothalamic feeding neurotransmitters. Neuron. 2003;40(6):1059–61. . 14687541 10.1016/s0896-6273(03)00809-2

[2] Williams KW, Elmquist JK. From neuroanatomy to behavior: central integration of peripheral signals regulating feeding behavior. Nature neuroscience. 2012;15(10):1350–5. doi: 10.1038/nn.3217 23007190 10.1038/nn.3217 PMC3763714

[3] Aponte Y, Atasoy D, Sternson SM. AGRP neurons are sufficient to orchestrate feeding behavior rapidly and without training. Nature neuroscience. 2011;14(3):351–5. doi: 10.1038/nn.2739 21209617 10.1038/nn.2739 PMC3049940

[4] Krashes MJ, Shah BP, Koda S, Lowell BB. Rapid versus delayed stimulation of feeding by the endogenously released AgRP neuron mediators GABA, NPY, and AgRP. Cell metabolism. 2013;18(4):588–95. doi: 10.1016/j.cmet.2013.09.009 . 24093681 10.1016/j.cmet.2013.09.009 PMC3822903

[5] Chen Y, Lin YC, Kuo TW, Knight ZA. Sensory detection of food rapidly modulates arcuate feeding circuits. Cell. 2015;160(5):829–41. doi: 10.1016/j.cell.2015.01.033 . 25703096 10.1016/j.cell.2015.01.033 PMC4373539

[6] Mandelblat-Cerf Y, Ramesh RN, Burgess CR, Patella P, Yang Z, Lowell BB, et al Arcuate hypothalamic AgRP and putative POMC neurons show opposite changes in spiking across multiple timescales. Elife. 2015;4 doi: 10.7554/eLife.07122 . 26159614 10.7554/eLife.07122 PMC4498165

[7] Adamantidis A, de Lecea L. Physiological arousal: a role for hypothalamic systems. Cellular and molecular life sciences: CMLS. 2008;65(10):1475–88. doi: 10.1007/s00018-008-7521-8 . 18351292 10.1007/s00018-008-7521-8 PMC11131926

[8] Cason AM, Smith RJ, Tahsili-Fahadan P, Moorman DE, Sartor GC, Aston-Jones G. Role of orexin/hypocretin in reward-seeking and addiction: implications for obesity. Physiology & behavior. 2010;100(5):419–28. doi: 10.1016/j.physbeh.2010.03.009 . 20338186 10.1016/j.physbeh.2010.03.009 PMC2886173

[9] Jennings JH, Rizzi G, Stamatakis AM, Ung RL, Stuber GD. The inhibitory circuit architecture of the lateral hypothalamus orchestrates feeding. Science. 2013;341(6153):1517–21. doi: 10.1126/science.1241812 . 24072922 10.1126/science.1241812 PMC4131546

[10] Celio MR. Calbindin D-28k and parvalbumin in the rat nervous system. Neuroscience. 1990;35(2):375–475. . 2199841 10.1016/0306-4522(90)90091-h

[11] Meszar Z, Girard F, Saper CB, Celio MR. The lateral hypothalamic parvalbumin-immunoreactive (PV1) nucleus in rodents. The Journal of comparative neurology. 2012;520(4):798–815. doi: 10.1002/cne.22789 . 22020694 10.1002/cne.22789 PMC3523738

[12] Markram H, Toledo-Rodriguez M, Wang Y, Gupta A, Silberberg G, Wu C. Interneurons of the neocortical inhibitory system. Nature reviews Neuroscience. 2004;5(10):793–807. Epub 2004/09/21. doi: 10.1038/nrn1519 . 15378039 10.1038/nrn1519

[13] DeFelipe J, Lopez-Cruz PL, Benavides-Piccione R, Bielza C, Larranaga P, Anderson S, et al New insights into the classification and nomenclature of cortical GABAergic interneurons. Nature reviews Neuroscience. 2013;14(3):202–16. doi: 10.1038/nrn3444 . 23385869 10.1038/nrn3444 PMC3619199

[14] Hu H, Gan J, Jonas P. Interneurons. Fast-spiking, parvalbumin(+) GABAergic interneurons: from cellular design to microcircuit function. Science. 2014;345(6196):1255263 Epub 2014/08/02. doi: 10.1126/science.1255263 . 25082707 10.1126/science.1255263

[15] Girard F, Meszar Z, Marti C, Davis FP, Celio M. Gene expression analysis in the parvalbumin-immunoreactive PV1 nucleus of the mouse lateral hypothalamus. The European journal of neuroscience. 2011;34(12):1934–43. doi: 10.1111/j.1460-9568.2011.07918.x . 22128821 10.1111/j.1460-9568.2011.07918.x

[16] Opland D, Sutton A, Woodworth H, Brown J, Bugescu R, Garcia A, et al Loss of neurotensin receptor-1 disrupts the control of the mesolimbic dopamine system by leptin and promotes hedonic feeding and obesity. Mol Metab. 2013;2(4):423–34. doi: 10.1016/j.molmet.2013.07.008 . 24327958 10.1016/j.molmet.2013.07.008 PMC3857883

[17] Ting JT, Daigle TL, Chen Q, Feng G. Acute brain slice methods for adult and aging animals: application of targeted patch clamp analysis and optogenetics. Methods in molecular biology. 2014;1183:221–42. doi: 10.1007/978-1-4939-1096-0_14 . 25023312 10.1007/978-1-4939-1096-0_14 PMC4219416

[18] Povysheva NV, Zaitsev AV, Gonzalez-Burgos G, Lewis DA. Electrophysiological heterogeneity of fast-spiking interneurons: chandelier versus basket cells. PloS one. 2013;8(8):e70553 Epub 2013/08/21. doi: 10.1371/journal.pone.0070553 . 23950961 10.1371/journal.pone.0070553 PMC3741302

[19] Keshavarzi S, Sullivan RK, Ianno DJ, Sah P. Functional properties and projections of neurons in the medial amygdala. The Journal of neuroscience: the official journal of the Society for Neuroscience. 2014;34(26):8699–715. doi: 10.1523/JNEUROSCI.1176-14.2014 . 24966371 10.1523/JNEUROSCI.1176-14.2014 PMC6608208

[20] Peng H, Bria A, Zhou Z, Iannello G, Long F. Extensible visualization and analysis for multidimensional images using Vaa3D. Nat Protocols. 2014;9(1):193–208. doi: 10.1038/nprot.2014.011 24385149 10.1038/nprot.2014.011

[21] Qi J, Zhang S, Wang HL, Barker DJ, Miranda-Barrientos J, Morales M. VTA glutamatergic inputs to nucleus accumbens drive aversion by acting on GABAergic interneurons. Nature neuroscience. 2016 doi: 10.1038/nn.4281 . 27019014 10.1038/nn.4281 PMC4846550

[22] Root DH, Mejias-Aponte CA, Zhang S, Wang HL, Hoffman AF, Lupica CR, et al Single rodent mesohabenular axons release glutamate and GABA. Nature neuroscience. 2014;17(11):1543–51. doi: 10.1038/nn.3823 . 25242304 10.1038/nn.3823 PMC4843828

[23] Aponte Y, Lien CC, Reisinger E, Jonas P. Hyperpolarization-activated cation channels in fast-spiking interneurons of rat hippocampus. The Journal of physiology. 2006;574(Pt 1):229–43. doi: 10.1113/jphysiol.2005.104042 . 16690716 10.1113/jphysiol.2005.104042 PMC1817792

[24] Livak KJ, Schmittgen TD. Analysis of relative gene expression data using real-time quantitative PCR and the 2(-Delta Delta C(T)) Method. Methods. 2001;25(4):402–8. doi: 10.1006/meth.2001.1262 . 11846609 10.1006/meth.2001.1262

[25] Petreanu L, Huber D, Sobczyk A, Svoboda K. Channelrhodopsin-2-assisted circuit mapping of long-range callosal projections. Nature neuroscience. 2007;10(5):663–8. doi: 10.1038/nn1891 . 17435752 10.1038/nn1891

[26] Atasoy D, Aponte Y, Su HH, Sternson SM. A FLEX switch targets Channelrhodopsin-2 to multiple cell types for imaging and long-range circuit mapping. The Journal of neuroscience: the official journal of the Society for Neuroscience. 2008;28(28):7025–30. doi: 10.1523/JNEUROSCI.1954-08.2008 . 18614669 10.1523/JNEUROSCI.1954-08.2008 PMC2593125

[27] Franklin KBJ, Paxinos G. Paxinos and Franklin's The mouse brain in stereotaxic coordinates. Fourth edition. ed Amsterdam: Academic Press, an imprint of Elsevier; 2013 1 volume (unpaged) p.

[28] Lintas A. Discharge properties of neurons recorded in the parvalbumin-positive (PV1) nucleus of the rat lateral hypothalamus. Neuroscience letters. 2014;571:29–33. doi: 10.1016/j.neulet.2014.04.023 . 24780564 10.1016/j.neulet.2014.04.023

[29] Shang C, Liu Z, Chen Z, Shi Y, Wang Q, Liu S, et al BRAIN CIRCUITS. A parvalbumin-positive excitatory visual pathway to trigger fear responses in mice. Science. 2015;348(6242):1472–7. doi: 10.1126/science.aaa8694 . 26113723 10.1126/science.aaa8694

[30] Wallace ML, Saunders A, Huang KW, Philson AC, Goldman M, Macosko EZ, et al Genetically Distinct Parallel Pathways in the Entopeduncular Nucleus for Limbic and Sensorimotor Output of the Basal Ganglia. Neuron. 2017;94(1):138–52 e5. doi: 10.1016/j.neuron.2017.03.017 . 28384468 10.1016/j.neuron.2017.03.017 PMC5439268

[31] Alcaraz F, Marchand AR, Vidal E, Guillou A, Faugere A, Coutureau E, et al Flexible Use of Predictive Cues beyond the Orbitofrontal Cortex: Role of the Submedius Thalamic Nucleus. The Journal of neuroscience: the official journal of the Society for Neuroscience. 2015;35(38):13183–93. Epub 2015/09/25. doi: 10.1523/JNEUROSCI.1237-15.2015 . 26400947 10.1523/JNEUROSCI.1237-15.2015 PMC6605447

[32] Watson TC, Cerminara NL, Lumb BM, Apps R. Neural Correlates of Fear in the Periaqueductal Gray. The Journal of neuroscience: the official journal of the Society for Neuroscience. 2016;36(50):12707–19. Epub 2016/12/16. doi: 10.1523/JNEUROSCI.1100-16.2016 . 27974618 10.1523/JNEUROSCI.1100-16.2016 PMC5157112

[33] Stamatakis AM, Van Swieten M, Basiri ML, Blair GA, Kantak P, Stuber GD. Lateral Hypothalamic Area Glutamatergic Neurons and Their Projections to the Lateral Habenula Regulate Feeding and Reward. The Journal of neuroscience: the official journal of the Society for Neuroscience. 2016;36(2):302–11. doi: 10.1523/JNEUROSCI.1202-15.2016 . 26758824 10.1523/JNEUROSCI.1202-15.2016 PMC4710762

